# Supplementary material for: Are carbon emissions trading and green financial instruments synergistic? -Comprehensive quantitative research based on content analysis
Source: PLoS One. 2024 Mar 7;19(3):e0298601. doi: 10.1371/journal.pone.0298601 (PMC10919601; doi:10.1371/journal.pone.0298601)
Supplement: S1 Data — (ZIP) [file pone.0298601.s001.zip › Data/地级市Z_F工作报告.zip!/░▓╗╒/│╪╓▌╩╨/└┤╫╘╣1⁄2╓┌║┼í╛┬φ┐╦╩2╛▌═°í┐_2014.docx]

各位代表：

　　现在，我代表市人民政府，向大会作工作报告，请予审议，并请市政协委员和其他列席人员提出意见。

**一、2013年工作回顾**

　　2013年，在省委、省政府和市委的坚强领导下，我们深入贯彻落实党的十八大精神，团结和依靠全市人民共同奋斗，较好地完成了市三届人大二次会议确定的目标任务。预计，全市生产总值465亿元，增长11%；财政收入83.58亿元，增长16.6%；固定资产投资457亿元，增长22%；城镇居民人均可支配收入23525元，增长10%；农民人均纯收入8944元，增长12%。经济社会发展继续保持稳中向好、结构趋优、民生改善、社会和谐的良好态势。

　　（一）承接产业转移示范区建设成效显著，工业经济不断壮大。以示范区建设为契机，加快工业发展，做强经济实力，全年完成工业投资240亿元、增长27%，占总投资的52.5%；新增规模工业企业80户，规模以上工业增加值140亿元、增长15.5%，增幅居全省前两位。区位优势加快凸显。重大基础设施项目建设加速，池州九华山机场建成通航，开通北京、上海、成都、广州4条航线；池州长江公路大桥试桩工程开工，济祁高速池州连接线西岸工程开工建设，望东长江公路大桥、东至——九江高速公路建设加快，新改建国省干线公路170公里。园区功能不断提升。全市园区完成基础设施及配套建设投入61亿元，新拉开框架50平方公里，新建标准化厂房260万平方米。江南产业集中区完成35平方公里起步区“七通一平”，引进亿元以上项目58个、总投资813亿元，建成投产项目20个，完成固定资产投资61亿元，实现财政收入3.05亿元。市开发区功能日趋完善。池州高新区和东至、青阳开发区扩区获批。首位产业和主导产业加快集聚。电子信息产业新引进项目23个，安芯电子科技2条GPP芯片生产线等10个项目建成投产，实现产值60亿元，增长83%，占规模工业产值比重同比提高2.2个百分点。战略性新兴产业实现产值80亿元，增长32%，增幅居全省第一，中恒天福泰动力40万台发动机等60个亿元以上大项目建成投产。科技创新进步明显。高新技术产业增加值50亿元，增长20%。新增省级高新技术产业基地1个、高新技术企业11家、省级企业技术研发机构10家。发明专利授权量增长180%、居全省第二，万人有效发明专利拥有量超过1.2件、居全省第六。云电子书包国际标准启动制订，新增省著名商标25件、名牌产品11个。东至、青阳荣获“全国科技进步先进县”称号。企业帮扶扎实有效。坚持盘活存量与引进增量相结合，推动企业做大做强，新增国有融资性担保公司注册资本金5.5亿元、市工投公司中小企业还贷周转金3000万元，推进7家企业引入战略投资者，吸引资金1.3亿元。中小微企业新增贷款32亿元；规模工业企业利润总额增长21%。

　　（二）文化旅游示范区建设取得突破，现代服务业发展提速。积极争取池州在国家皖南国际文化旅游示范区规划中的核心定位，坚持政府主导、市场主体，综合实施景区景点打造、旅游环线建设、住宿餐饮配套、特色商品开发等项目，扎实推进示范区建设，全年服务业增加值170亿元，增长10%。旅游文化产业发展取得突破。有力保障了“地藏菩萨圣像开光活动”安全、有序、圆满，大愿文化园正式开园后四个月共接待游客60万人次。杏花村文化旅游区完成总体规划，起步区初见雏形，成为吸引市场投资的新载体，“杏花古村”等3个项目签约落户。齐山——平天湖等3个景区创建4A景区、九华天池景区创建国家生态旅游示范区通过验收。开业运营4家星级宾馆和6家经济型、快捷式酒店。新增省级休闲农业与乡村旅游示范县1个、四星级以上农家乐12个、地方特色旅游商品品牌生产销售企业20家，乡村旅游接待游客量达到总量的30%以上。制定实施旅游市场营销、机场航线培育政策，举办旅游营销推介活动70场（次），兑现奖补资金950万元。全年接待国内外游客3441万人次，旅游收入345亿元，分别增长30.3%、30.2%；新增省民营文化百强企业3家，文化产业增加值15亿元、增长25%。金融活力进一步增强。全市社会融资总量120亿元，其中新增信贷投放70亿元。农商行、村镇银行实现县区全覆盖，九华农商行启动第三次增资扩股。引进设立3家创投基金和2家金融服务公司，市开发区10亿元企业债券成功发行，贵池区9.5亿元、江南产业集中区15亿元企业债券分别获批和待批，九华股份基本完成上市发审程序，8家企业启动“新三板”、“四板”挂牌。现代服务业态不断丰富。制定商贸活市战略的实施意见和激励政策，重点支持电子商务、会展经济、品牌建设等领域，新增限额以上流通企业49户，社会消费品零售总额142亿元、增长14.5%。贵池区获批省级服务业综合改革试点。站前区入驻总部经济项目4个、楼宇经济和电子商务企业98家。全省首家市级电子商务协会正式成立。国际会展中心主体完工。

　　（三）生态文明示范区建设步伐加快，城乡统筹扎实推进。在全省率先成功创建国家森林城市，中国人居环境奖创建通过考核验收，国家生态市、环保模范城市规划通过国家评审。城市功能不断完善。实施市本级政府性投资项目73个，完成投资16.6亿元。新改建城市道路10公里、排水（雨水）管网26公里，维修改建城市桥梁5座，整治老旧小区2个。市文化馆、图书馆、科技馆、博物馆建成开放。国家绿色生态示范城区综合开发有序推进。“数字池州”电子政务公共云平台基本建成。市容市貌、交通秩序管理不断加强。主城区房产开工、销售面积分别增长17.5%、16.5%。农业农村经济平稳发展。坚持规划引领、整合资金，推动基础设施、公共服务向中心镇、中心村集中，促进农村人口有序转移，加快农业规模化经营。农村土地“三权”确权登记率97%以上，新增土地流转面积5.6万亩。新增规模以上农产品加工企业27家、家庭农场103个、省级专业合作社示范社5个、“三品”认证25个。建设部级水稻、油菜万亩示范片24个，新增省级现代农业示范区1个，新建蔬菜基地2000亩，新建和改造无性系良种茶园1万亩，粮食总产65.2万吨。兑现强农惠农政策补贴资金7.7亿元。农产品质量安全监管和禽流感等重大动植物疫病防控扎实有效。35个村整村推进扶贫开发投入4615万元。美好乡村和农村基础设施建设扎实推进。完成83个中心村规划编制，建成26个美好乡村重点示范村。农村危房改造10790户。开工建设19座水库除险加固、7处中小河流治理、2处大中型泵站更新改造工程，解决14.5万农村人口饮水安全问题，完成水利投资6.3亿元。县乡公路升级改造106公里，农村公路危桥加固改造68座。新增110千伏变电站3座，农村电网改造2416公里。完成农村土地整理4.08万亩，建设高标准基本农田13.26万亩，开发复垦新增耕地8172亩。生态保护建设力度加大。节能减排完成省定目标。东至、石台、青阳三县全部建成省级生态县。城市湿地森林公园完成造林700亩，清溪河、平天湖水环境综合治理取得阶段性成效，城东污水处理厂改造完成。升金湖管理体制机制不断完善，生态保护和开发利用有序推进。完成可再生能源建筑应用示范项目62个。人工造林8.9万亩，治理水土流失面积15平方公里。完成地质灾害隐患避让搬迁治理工程8处，实施“矿山复绿”治理5家，整合减少矿权17个，整治关闭非煤矿山27家、小煤矿5家。

　　（四）招商引资和扩大开放力度加大，市场主体活力明显增强。制定实施加强招商引资和发展民营经济的政策措施，全年民间投资320亿元，增长20%。招商引资成效明显。突出主导产业招商、产业链招商、全领域招商、以商招商、领导带头招商，深化重点区域招商合作，成立市直六个产业招商组，加强培训、宣传，提升招商能力，营造招商氛围。全年引进亿元以上项目130个，建成投产项目238个，全年招商项目到位资金360亿元、增长20%。央企合作项目新开工15个，知名民营企业合作项目新签约64个。发展环境不断优化。加快转变政府职能，开展两轮行政审批项目清理，市级行政审批项目减少286项，进驻政务中心196项、占总数的95%，项目平均办理时限压缩6个工作日，减、缓、免、降各类收费64项；加强土地、岸线、广告等公共资源管理，制定政府性投资项目、招投标交易等全过程规范管理制度，营造了外来资本、民间资本、本土企业公平公正、竞争有序的市场环境。民营经济发展加快。坚持放开搞活，市本级从2013年起连续5年，每年安排5000万元专项资金，重点为民营企业提供融资担保扶持。发放小额担保贷款2.01亿元，青年（大学生）创业园孵化82家创业企业，全市培育创业实体7419户，净增私营企业1686户、个体工商户4900户，1家民企进入全省企业百强，民营经济增加值占生产总值比重同比提高2个百分点。外经外贸稳步增长。进出口总额4.2亿美元，实际利用外资2.6亿美元，均增长22%。市开发区综合保税仓库建成使用。池州港口岸核心能力建设通过国家验收。

　　（五）民生保障和公共服务持续加强，人民群众幸福指数不断提高。坚持压缩一般支出，将支出重点和新增财力优先保障基本民生和公共服务，民生类支出99.7亿元，增长18.7%，占财政总支出的76.3%。民生工程扎实实施。33项民生工程和9件惠民实事完成投入27.55亿元，增长20.7%。发放医疗、孤儿、贫困生、贫困残疾人、生活无着人员等各类补助救助资金8.72亿元，实施农村清洁工程、“一事一议”奖补等各类工程项目579个，投入工程项目后续管养资金6279万元。就业和社会保障体系逐步健全。组织多层次创业、就业技能培训1.18万人，新增城镇就业1.8万人，转移农业劳动力1.96万人，城镇登记失业率控制在3.6%以内。城乡居民养老保险参保81.5万人，新农合和城镇居民医疗保险参保147.8万人，企业退休人员基础养老金人均月增149元，20种大病医疗报销比例上调15个百分点，医疗保险异地就医实现省内即时结算。城乡低保人均月补差326元和148元。建立普惠制高龄老人津贴制度，五保老人供养标准提高171元以上，新增社会养老服务床位1500张。实施主城区棚户区改造项目4个，保障性住房开工17935套、基本建成16689套，廉租住房租赁补贴发放5588户。社会公共服务不断加强。新改建幼儿园40所，建设义务教育标准化学校78所，改造农村薄弱学校24所，新增1所省示范高中。县级公立医院综合改革全面推进，乡村医生养老保障制度初步建立，新农合住院实际补偿比提高1个百分点，市第三人民医院主体工程完工，乡镇卫生院新改建13所。完成838个广播电视村村通、20个公共文化服务信息化项目建设。成功举办第三届全国绿运会、国际铁人三项赛等重大赛事。《池州市志》完成编纂。第三次全国经济普查扎实开展。人口计生工作继续保持在全省先进行列，人口自然增长率4.26‰。妇女、儿童、老年人和残疾人事业加快发展，审计、统计、物价、供销、气象、地震、民族、宗教、对台、外事、侨务、档案、保密、地方志、新闻出版、社会科学等各项工作不断加强。

　　（六）社会管理科学化水平不断提升，和谐池州建设深入推进。坚持理顺关系、重心下移，不断加强村和社区基层自治组织建设，促进村（居）民自我管理、自我教育、自我服务、自我监督，夯实社会管理基础。社区等基层服务平台逐步健全。按照为民服务统一进社区办理的目标，启动实施主城区标准化社区创建“三年计划”，推进就业、社保、综治、计生等政府公共服务项目进入社区平台，吸引社会力量积极发展家政、物业、中介等市场化服务，就近就地为居民服务，建立市场化服务日常监管和社区工作居民满意评价机制，建成标准化社区11个。乡村为民服务全程代理机制进一步推进，村务公开民主管理继续深化。平安建设深入开展。依法治市加快推进，“六五”普法通过省中期督查。信访秩序规范有序，社会治安防控体系不断健全，人民群众安全感和满意度测评得分居全省前列。安全生产形势持续稳定好转。质量强市活动扎实开展，食品药品安全监管持续加强。科学防范和应对突发事件能力不断提高。精神文明建设深入推进，国防动员、人民防空和民兵预备役等工作成效明显，争创省文明城市“三连冠”取得良好成绩，创建省双拥模范城实现“四连冠”。

　　在推进经济社会又好又快发展的过程中，我们高度重视加强政府自身建设，坚持依法行政，推进管理创新，加强效能建设，坚持廉洁自律，树立为民、务实、清廉良好形象。主动接受人大的法律监督、工作监督和政协的民主监督，注意听取各民主党派、工商联、无党派人士和各人民团体的意见，办理人大代表议案、建议147件和政协委员提案311件。加大政务公开和政府信息公开力度，开通政务微博，回复办理市长信箱、市长热线、网民留言1912条。认真贯彻落实作风建设和廉洁从政各项规定，健全廉政风险防控和处置长效机制，强化政府投资、经济责任、公共财政绩效等审计监督，政风建设取得新成效。

　　各位代表！过去的一年，发展历程艰辛，成绩来之不易。这是省委、省政府和市委坚强领导，市人大、市政协和社会各界鼎力支持，全市人民团结奋斗、共克时艰的结果。在此，我代表市人民政府，向全市人民，向广大工人、农民、知识分子、干部、驻池解放军指战员、武警消防官兵和公安政法干警，向各民主党派、各人民团体、社会各界人士和各位老领导、老同志，致以崇高的敬意!向关心、支持池州建设的中央和省驻池单位，向在我市创业的境内外投资者、建设者，向给予市政府高度信任和倾力支持的各位人大代表、政协委员，表示衷心的感谢!

　　在总结成绩的同时，我们也清醒地看到存在的问题和不足，主要是：宏观经济下行压力加大，产业基础薄弱的状况还没有得到根本改变，县域经济实力仍然较弱，做大经济总量与转型发展同步推进的难度加大，财政增收的制约因素增多；社会事业发展相对滞后，公共服务供给与民生改善需求差距仍然较大，社会治理基础仍较薄弱；政府职能转变需要进一步加快，运用市场化的手段推动发展的能力需要进一步提升，发展环境需要进一步优化，等等。对此，我们必须高度重视，坚持“两点论”，运用“底线思维”，从最关键、最薄弱、最基础的环节抓起，逐步加以解决。

**二、2014年工作安排**

　　今年是全面贯彻落实党的十八届三中全会精神、全面深化改革的第一年，是实现“十二五”规划目标的关键一年。当前，各项改革红利不断释放，国家提出建设长江经济新的支撑带，我市“三区”建设战略平台效应更加凸显，经济社会发展具备良好的外部条件和内部支撑。同时，国内外经济环境错综复杂性呈现常态化，给我们的工作带来一系列困难和挑战。必须牢牢抓住重大战略机遇，充分做好应对各种复杂情况的准备，坚定信心，积极作为，提质提效，奋力把经济社会发展提高到一个新的水平。

　　今年政府工作的总体要求是：全面贯彻落实党的十八大和十八届二中、三中全会精神，以全面深化改革为动力，以开展群众路线教育实践活动为契机，以推进“三区”发展为主线，以幸福池州建设为目标，坚持稳中求进的总基调，突出工业、旅游两大产业支撑，突出人居城市、新型城镇化、美好乡村三大重点建设，着力加快转型升级，着力激发市场活力，着力保障改善民生，保持经济持续健康较快发展、社会和谐稳定。

　　综合考虑，今年经济社会发展的主要目标是：全市生产总值增长11%，财政收入增长13%，固定资产投资增长18%，规模以上工业增加值增长15%，社会消费品零售总额增长14%，进出口总额增长15%，城镇居民人均可支配收入增长12%，农民人均纯收入增长12%，城镇新增就业1.44万人，城镇登记失业率控制在4.5%以内，人口自然增长率控制在7‰以内，单位GDP能耗下降率和主要污染物减排量达到省控目标。

　　（一）坚持转型发展，加快推动工业经济优化升级

　　进一步加快皖江城市带承接产业转移示范区建设，大力发展“两高一首”产业，在工业强市、转型发展上取得新成效。全年战略性新兴产业产值增幅高于全省平均水平，高新技术产业增加值占规模工业增加值的比重达到全省平均水平。着力提升产业层次。紧盯产业链高端，突出关键环节，强化上下游配套，大力推进招商引资，严格落实各类开发区建设项目投资强度、容积率和建筑密度、预期亩均税收等标准，首位产业力争新引进项目40个、建成投产20个、总产值突破100亿元。加快战略性新兴产业集聚发展，推进传统产业技术改造和产品升级换代，积极引进战略投资者推进生产要素流动和重组，重点加快80个亿元重大工业项目建设。完成工业固定资产投资275亿元，其中技改投资140亿元；新增规模工业企业60户。推动开发区转型升级。制定各类开发区产业发展指导目录，进一步提升开发区主导产业定位，推进市开发区、池州高新区提高首位产业集中度，合理布局生产、生活用地；推进前江工业园加快向材料深加工发展、今后禁止新上冶炼项目，东至经济开发区由化工向生物医药材料产业升级，大渡口经济开发区向现代服务业发展，青阳经济开发区由机械加工向装备制造产业转型，主导产业集中度提高5个百分点以上。加快江南产业集中区工业首发区、梅龙中心城建设，引进亿元以上产业项目80个，建成投产25个，完成固定资产投资70亿元、财政收入4亿元，拉动全市经济增长1个百分点。加快推进科技创新。深入开展产学研对接，引导创新要素向企业集聚，加强市开发区、池州高新区科技孵化器等创新平台建设，支持江南产业集中区争创国家级高新技术产业开发区。新增省级高新技术企业10家、省级企业技术研发机构8家，发明专利申请量、授权量分别增长20%以上，创建省著名商标10件、名牌产品5个。创新人才激励保障机制，有效吸引和留住高层次创新创业人才。

　　（二）坚持特色发展，大力拓展提升旅游等现代服务业

　　围绕推进实施即将出台的皖南国际文化旅游示范区规划，在建设世界佛文化观光胜地和国际生态休闲城市上迈出新步伐，加快发展以旅游文化为龙头的第三产业，全年接待国内外游客4056万人次、旅游收入412亿元，分别增长20%、22%；服务业增加值增长10%以上。加快完善旅游综合交通体系。力争九华山机场启动国际直航包机和航线申报，全年旅客吞吐量15万人次以上。开工建设池州长江公路大桥，完成宁安城际铁路池州段、济祁高速连接线建设，推进望东长江公路大桥、东至—九江高速、池州港旅游口岸等项目建设，加快九华山旅游环线、高岭—牛头山、东流—尧渡、青阳—木镇、香源线二期、贵铜公路改线等国省干线建设，推进池州—九华山—黄山城际、池州—九江城际、殷汇—石台高速、过江通道、市域轨道交通等项目前期工作，力争启动鹅毛洲右汊航道、青通河、秋浦河、九华河等长江干支流航道整治工程。完成交通投资40亿元。着力打造旅游目的地品牌。进一步发挥九华山的龙头带动作用，以游客需求为导向，大力引进市场主体，重点推进大愿文化园“九华大典”、杏花村文化旅游区“杏花古村”等20个5亿元以上项目建设，加快九华山申报世界地质公园步伐，提升齐山—平天湖风景区、城市湿地森林公园、升金湖的旅游功能，建成一批经济型、快捷式酒店和停车场、旅游购物品牌连锁店，开发一批地方特色旅游商品。加大景区品牌创建力度，推进乡村旅游发展，启动石台牯牛降5A级景区创建，新增4A级景区1家、3A级景区4家、国家生态旅游示范区1家、4星级以上农家乐10家。开展旅游系列宣传营销活动，提升整体营销策划和宣传水平，建立景区、景点、机场、酒店、农家乐、旅行社等利益共享合作机制，推动市场主体之间联动互补发展。加快文化旅游体育融合发展，文化产业增加值增长20%。大力发展新兴服务业态。进一步落实商贸活市战略政策措施，新增亿元流通企业2家、限额以上流通企业40户、千万美元进出口实绩企业3家以上。加快发展现代物流业，依托机场、高速、铁路、港口等综合交通枢纽，加快建设以配套园区产业发展为重点的综合物流园，积极培育航空物流，大力引进第三方物流企业。推进站前区电子商务园区建设，积极创建电子商务运营平台，大力发展电子商务。加快培育会展企业，推进国际会展中心等会展基础设施和服务体系建设。大力培育各类咨询、评估、认证、代理等中介服务机构，规范中介服务市场。

　　（三）坚持统筹发展，扎实推进美丽池州建设

　　以创建国家生态文明建设先行示范区为契机，深入实施《关于加快推进生态文明建设的决定》，在机制创新和品牌创建上跨上新台阶，加快打造城市品牌，推进城乡一体化发展，增强城市影响力和集聚力。加快建设生态宜居城市。市本级安排政府性投资项目87项，年度计划投资24.5亿元。进一步完善城市综合交通、排水除涝等专项规划，新改建城市主次干道13.5公里、雨污分流管网27公里、燃气管线95公里。深入实施清溪河、平天湖水环境综合治理工程。推进清溪污水处理厂升级改造。谋划推进主城区池口片长江岸线整治工程，加快实施新港新湖等22个棚户区改造项目。推进天堂湖绿色生态示范城区建设，新改建绿色建筑90万平方米。优先发展公共交通。加快“数字池州”建设，实施城市精细化管理，不断提升城市品质。大力发展生态产业，重点培育居住和休闲养生业态。加快推进新型城镇化。抢抓国家推进新型城镇化战略机遇，围绕“人的城镇化”新理念，推进东至、青阳两县的省新型城镇化试点工作。实施新一轮市域及县域城镇体系规划与修编，开展县城规划建设管理水平提质达标行动，保护自然山水，留住城市记忆，优化空间布局；以产业发展为依托，促进中心城市、县城和中心城镇合理分工、功能互补、协同发展，推进产城一体、融合发展；加强城镇建设用地集约利用，积极探索引进社会资本参与城镇公用设施投资运营，提高城镇建设和管理水平；深化户籍制度改革，全面推行居住证制度，自愿、分类、有序推进农业转移人口市民化，解决3万农业转移人口落户城镇问题。加快建设美好乡村。调整完善美好乡村建设布点与规划，有效整合各类涉农项目资金，重点建设34个中心村，完善以垃圾污水处理、村庄道路等为重点的基础设施和基本公共服务，逐步建立管护长效机制。开工建设5处中小河流治理、2座排涝站更新改造、19座小型水库除险加固工程，启动实施小型水利工程改造提升“5588”行动计划，建设高标准基本农田17.6万亩，解决10万农村人口饮水安全问题，完成水利投资5亿元以上。升级改造县乡公路80公里、新一轮农村电网1000公里，加固改造农村公路危桥60座。加强气象防灾减灾体系建设。继续做好整村推进扶贫开发工作。加快推进农业现代化。稳步推进农村综合改革示范试点，促进土地承包经营权流转健康发展，加快建立新型农业经营体系，推进农业规模化经营。新增土地流转面积5万亩，引进5个5000万元以上农产品深加工项目，新增省市级龙头企业20家，发展省市级农民专业合作社示范社20个、家庭农场80个，新增“三品”认证10个。扎实推进省级现代农业示范区和农业产业化示范区建设，大力发展地方特色优质农产品，加强品牌整合，粮食总产稳定在65万吨以上，新建和改造无性系良种高效茶园1万亩。加强“菜篮子”工程体系建设，引导蔬菜基地产品优先供应本地市场。加强基层农技推广服务、动植物疫病防控和农产品质量安全检测监管。加快生态文明建设。加强生态文明制度建设，划定生态保护红线，确定各类风景名胜区、自然保护区保护与开发边界，落实用途管制。加快国家生态市、国家环保模范城市、全国低碳城市试点创建，确保市辖三县全部建成国家生态县。推进升金湖国家级湖泊生态环境保护试点项目。强化大气、水体监测，优化主城区能源结构，加强饮用水源地保护，启动应急备用水源建设规划。推进千万亩森林增长工程，人工造林7.3万亩。整顿关闭非煤矿山36家。

　　（四）坚持创新发展，全面深化重点领域和关键环节改革

　　紧紧围绕使市场在资源配置中起决定性作用，在深化各项改革、创新体制机制、优化发展环境上实现新突破。深化行政体制改革。大力推进行政审批制度改革，承接落实好中央和省取消、下放的审批事项，今年再减少市级行政审批事项50项以上，对保留的审批事项除涉及和影响国家公共安全以外的全部进政务中心办理，办理程序向社会公开、接受社会监督并不断改进。制定地方政府职能转变和机构改革实施方案，按照自上而下的原则，深化行政执法体制改革，完成工商、质监行政管理体制调整和食品药品监管体制改革，提高食品药品、安全生产、环境保护、劳动保障、城市管理等领域执法和服务水平。推进事业单位分类改革。加快行业协会商会和中介服务组织与行政机关真正脱钩。制定政府购买服务具体实施办法。完善国有资产资金和公共资源管理机制。建立健全政府性资金和债务全口径预算管理体系，严格使用标准和程序，加强政府性投资项目、专项资金运行的绩效评估。继续推进国有房产、土地、矿产、岸线、采砂、广告、保障性住房、特许经营等资产资源配置管理规范化，实行标准公开、程序公开、市场竞争配置，进一步加强招投标交易管理，健全决策、执行、监督相分离的管理体制机制，创造公开、公平、公正的市场环境。推进其他各项改革。继续做好“营改增”扩围试点。巩固和完善县级公立医院综合改革。建立健全更加公平可持续的社会保障制度，整合城乡居民基本养老、基本医疗保险制度，加快建立符合实际的住房保障标准体系。统筹推进教育、文化、科技、就业创业、收入分配等各项改革。

　　（五）坚持开放发展，切实增强经济发展活力

　　把招商引资作为推动“三区”发展的第一要事，更好地发挥金融对转型发展的支撑作用，充分激发创新创业创造活力，掀起新一轮民营经济大发展的热潮。推进招商工作提质提效。聚焦“两高一首”产业，立足规划布局，招真商、引实资、聚人才，确保全年引进项目410个，投产项目250个，到位资金420亿元、增长16%；实际利用外资3亿美元，增长15%。坚持领导靠前招商，强化主要领导招商工作负责制，建立招商项目全程跟踪服务和领导联系调度制度。完善招商引资绩效考核评价体系。提升金融支撑力。社会融资总量130亿元，其中新增信贷投放80亿元以上。积极跟进股票发行注册制改革和“新三板”扩容，运用企业债、中期票据、融资租赁、资产证券化等市场化融资方式，推进市工投公司按市场机制参股基金，支持企业与各类创投基金合作实施股份制改造，登陆多层次资本市场，力争九华股份上市发行、6家企业进入“新三板”、“四板”挂牌融资。加强与政策性金融机构的战略合作，大力引进股份制银行、基金、证券及融资租赁等域外金融机构，力争交通银行池州分行开业运营，推进九华农商行、融资性担保公司增资扩股。大力发展普惠金融，引导银行业金融机构适应利率市场化改革，丰富和创新“三农”、中小微企业金融服务方式。引导符合条件的民间资本发起设立金融租赁公司、消费金融公司等民营金融机构。积极防范金融风险。大力发展民营经济。坚持权利平等、机会平等、规则平等，推进民营企业等各类市场主体依法平等使用生产要素、公平公正参与市场竞争。搭建民营经济发展服务平台，加强分层分类帮扶指导，推进项目、资本、技术等资源要素对接，认真落实行政事业性收费减免等减负政策，净增私营企业1500户、个体工商户5500户。完善创业扶持政策，实施公司注册资本登记制度改革，加强各类创业园、创业市场、孵化基地建设，推进小额担保贷款整贷直发，形成政府激励创业、社会支持创业、劳动者勇于创业的新机制，新增省级小微企业创业基地1个，创业培训2300人，培育创业实体7000户。

　　（六）坚持为民发展，不断提高民生改善和社会治理水平

　　按照守住底线、突出重点、完善制度、引导舆论的思路，做好民生保障和改善工作，夯实社会治理基层基础，确保人民安居乐业、社会安定有序。提升民生工程实效。坚持积极而为、量力而行，上下同步、协调一致，突出保障基本民生，组织实施省33项民生工程和市10件惠民实事，强化民生工程项目绩效和运行质量管理，推进分类管养，提高民生工程质量效益和群众满意度。加强就业和社会保障。进一步完善创业带动、政策帮扶、城乡统筹“三位一体”的就业服务体系，依托产业发展创造更加充分、更高质量的就业机会，开展就业技能培训9000人，政府补助提供就业岗位1万个。进一步扩大城乡居民社会养老保险覆盖面，加强城乡最低生活保障动态管理，做好医疗救助、孤儿救助、临时救助工作。建成市残疾人综合服务中心。加强城乡养老服务体系建设，鼓励和支持社会力量参与兴办养老服务产业和居家养老服务，建成市老年公寓。新开工保障性住房28242套，基本建成15903套，发放低收入家庭住房补贴5000户，规范公共租赁住房管理，确保公共资源公平善用。强化基层基础服务功能。继续开展主城区标准化社区建设，完善社区平台服务功能和运行机制，把社区工作考核权交给社区居民，实现政府治理和社会自我调节、居民自治良性互动。完善村民自治制度，深化村务公开民主管理。积极培育和规范发展社会组织，倡导发展志愿者队伍，吸引更多社会力量参与社会管理和服务。加快发展社会事业。启动实施第二期学前教育三年行动计划，创建义务教育标准化学校70所，提升职业教育服务产业发展能力，促进城乡、区域教育和各级各类教育协调发展。巩固基本医保覆盖面，推进乡镇卫生院和社区卫生服务能力建设，建成运营市第三人民医院，提高基本公共卫生服务水平。大力引进社会资本创办医疗机构。加强血防工程建设，抓好重大传染病和职业病防控。深入实施文化惠民工程，加强文物、非物质文化遗产保护和开发利用，推进第一次全国可移动文物普查。巩固第三届安徽省文明城市创建成果，广泛开展精神文明创建活动。出版发行《池州市志》。实施全民健身计划，积极发展体育产业。加强人口计生工作，提高出生人口素质。加强统计基层基础，完成第三次全国经济普查。加强外事、侨务、对台、物价、供销、气象、保密、档案、地方志、防震减灾、新闻出版、社科研究等各项工作，积极发展红十字和福利慈善事业。深化平安池州建设。坚持依法治市，深入开展“六五”普法，加强法律援助工作。不断完善社会治安防控体系，严厉打击违法犯罪活动。完善人民调解、行政调解、司法调解联动的工作体系，加强社会矛盾纠纷排查化解，依法维护群众合法权益。坚持重大事项社会稳定风险评估，加强网络舆情监测处置和正面引导。健全公共安全体系，完善各类应急预案和处置流程，提高防灾减灾和应对突发事件能力。严格落实安全生产责任制，加强食品安全和产品质量监管。巩固超限超载治理成果，推进科技治超。依法维护民族团结和宗教和谐稳定。积极支持驻池部队建设，加强国防教育、国防动员、民兵预备役和人民防空工作，巩固双拥模范城创建成果。

**三、加强政府自身建设**

　　抢抓发展机遇，应对发展挑战，实现发展目标，必须全面加强政府自身建设，不断提高政府的公信力、执行力和效能，加快建设法治政府和服务型政府。

　　（一）扎实开展群众路线教育实践活动。按照中央和省委、市委统一部署，深入开展以为民务实清廉为主要内容的群众路线教育实践活动，认真抓好“学习教育、听取意见，查摆问题、开展批评，整改落实、建章立制”三个环节，着力解决“四风”方面存在的突出问题，确保活动取得实实在在的成效。

　　（二）切实增强服务发展和服务群众能力。认真学习贯彻党的十八届三中全会精神，切实提升把握发展规律、运用市场机制、驾驭复杂局面、解决实际问题的能力；涉及社会公共利益、群众切身利益的工作，都要广泛征求不同层面、不同角度的意见和建议，建立群众广泛参与决策和监督的长效机制，办好对当前有用、对长远有利的事，让发展成果更多更公平惠及全体人民。

　　（三）着力提升依法行政和有效治理水平。完善政府重大决策规则和程序，严格按照法定权限和程序履行职责。自觉接受人大法律监督、工作监督，主动接受政协民主监督，认真办理人大代表议案、建议和政协委员提案，诚恳听取各民主党派、工商联、无党派人士和人民团体的意见和建议，积极支持工会、共青团、妇联开展工作。全面正确履行政府职能，推行“正面标杆”和“负面清单”的管理模式。严格绩效管理，强化工作责任，加强督查问责，提升工作效能，推进工作高效落实。加强政务公开和政府信息公开，接受群众监督、舆论监督、网络监督。

　　（四）牢固树立清正廉洁的良好形象。认真落实中央和省、市关于改进作风的各项规定，严格执行党风廉政建设责任制，坚持用制度管权管事管人，强化领导干部经济责任审计，加大行政监察和审计监督力度，坚决纠正损害群众利益不正之风。认真落实党政机关厉行节约反对浪费等各项条例规定，压缩“三公”经费，把有限的财力用在支持发展、改善民生上。加强对政府班子自身和公务员队伍的教育、管理和监督，努力实现干部清正、政府清廉、政治清明。

　　各位代表！改革时不我待，发展永无止境。让我们紧密团结在以习近平同志为总书记的党中央周围，在省委、省政府和市委的坚强领导下，解放思想，凝聚力量，锐意进取，攻坚克难，奋力推进“三区”大发展，为加快建设经济繁荣、环境优美、社会和谐、生活富裕的幸福池州而努力奋斗！
